# Predicting 30-day readmission using DRG-based hospitalization data: a large real-world logistic regression model from a tertiary hospital

**DOI:** 10.3389/fpubh.2026.1776878

**Published:** 2026-02-25

**Authors:** Wei Shao, Lixin Shu, Xufang Wang, Fei Yu, Ting Zhou, Dan Han

**Affiliations:** 1 Department of Pharmacy, Liaoning Institute of Basic Medical Sciences, Shenyang, China; 2 School of Pharmacy, Naval Medical University, Shanghai, China; 3 Department of Pharmacy, Second Affiliated Hospital of Chongqing Medical University, Chongqing, China

**Keywords:** 30-day readmission, DRG, hospital performance, logistic regression, prediction model, real-world data

## Abstract

**Background:**

Early unplanned readmission is a key quality indicator in Diagnosis-Related Groups (DRG)–based payment systems. Despite China’s rapid expansion of DRG reform, evidence on hospital-wide predictors of 30-day readmission using large-scale real-world data from tertiary hospitals remains limited. This study developed and evaluated a DRG-based logistic regression model for predicting 30-day readmission.

**Methods:**

We conducted a single-center retrospective study using administrative hospitalization data from a high-volume tertiary hospital in Shanghai, China. We extracted 65,215 inpatient episodes from the hospital (January 2023–December 2024). After excluding discharges in December 2024 due to incomplete follow-up (*n* = 3,109), 62,106 admissions were retained to estimate the overall readmission rate. For multivariable modeling, 21 additional cases with missing DRG variables were removed, yielding 62,085 complete observations. Predictors included age, length of stay, total cost, discharge year, and major DRG categories. Total hospital cost was modeled in its original unit (1 Chinese Yuan) to preserve the raw scale of administrative reporting; however, for interpretation, marginal effects per 1,000 CNY increase were also calculated. Model performance was evaluated using the area under the ROC curve (AUC), Brier score, Hosmer–Lemeshow test, and a decile-based calibration plot.

**Results:**

The 30-day readmission rate was 13.0%. In unadjusted comparisons, patients who were readmitted had shorter median hospital stays (3 vs. 4 days) and lower total costs. After multivariable adjustment, longer length of stay was associated with increased readmission risk (OR 1.016 per day, *p* < 0.001), while total cost showed a statistically significant but small association (*p* = 0.003). Age and discharge year were not significant predictors. DRG major categories had a strong overall association (global *p* < 0.001). The model showed moderate-to-good discrimination (AUC = 0.743) and acceptable overall accuracy (Brier score = 0.098), with visually adequate calibration despite a statistically significant Hosmer–Lemeshow test.

**Conclusion:**

Using comprehensive DRG-based real-world data, we developed an interpretable prediction model for 30-day readmission with moderate-to-good discrimination and acceptable calibration. Clinical case-mix captured by DRG categories and patient-level complexity reflected by longer length of stay were key determinants of early readmission. The model may support risk stratification, quality improvement, and performance monitoring in DRG payment environments. The findings may also inform policy discussions on aligning DRG efficiency incentives with patient safety outcomes.

## 1 Introduction

Hospital readmission within 30 days has long been recognized as a key indicator of healthcare quality, continuity of care, and system efficiency (1, 2). Reducing preventable readmissions has become a strategic priority across health systems worldwide (3), given their association with adverse clinical outcomes, increased service utilization, and substantial financial pressures on providers and payers (4, 5). In settings that employ Diagnosis-Related Group (DRG)–based prospective payment, 30-day readmission rates carry even greater policy relevance because they reflect the balance between cost containment, discharge practices, and the quality of post-acute transitions.

Since 2019, China has rapidly expanded the adoption of DRG-based prospective payment with the overarching aim of improving efficiency and promoting value-based healthcare delivery (6, 7). However, evidence describing readmission patterns and their determinants in Chinese tertiary hospitals remains limited. Most existing analyses have been disease-specific, focused on single departments, or restricted by small sample sizes. Few studies have leveraged large-scale, real-world DRG datasets to systematically examine factors associated with short-term readmission across diagnostic categories (8). Moreover, robust prediction models are scarce; those that exist rarely include essential performance indicators such as discrimination, calibration, and overall predictive accuracy, and often omit calibration assessments entirely (9). Conceptually, DRG-based payment may influence hospital behavior through financial incentives that prioritize efficiency and cost containment. Under prospective fixed reimbursement, hospitals may face pressure to reduce length of stay (LOS) in order to maintain financial margins. However, excessively shortened LOS may increase the risk of unresolved clinical instability at discharge, potentially leading to early unplanned readmission. Conversely, prolonged LOS within a given DRG may reflect greater clinical complexity or complications, which may also elevate readmission risk. Therefore, LOS may operate as both an efficiency indicator and a proxy for clinical severity, depending on the analytic level. Understanding this interaction is essential for interpreting readmission patterns within DRG systems. Recent Chinese multi-center evaluations have begun to examine the impact of DRG-based payment reform on hospital behavior, cost containment, and efficiency indicators (10, 11). Several provincial pilot studies have reported reductions in average length of stay and stabilization of inpatient expenditure following DRG implementation, though evidence regarding quality-related outcomes—particularly readmission—remains limited. Most existing Chinese studies have focused on financial performance metrics rather than risk-adjusted patient-centered outcomes, highlighting the need for hospital-level analyses integrating DRG classification and readmission monitoring.

To address these gaps, we used real-world administrative inpatient data from a large tertiary hospital in China to develop a DRG-based prediction model for 30-day readmission. After excluding discharges without complete follow-up information and conducting a complete-case analysis, a total of 62,085 hospitalizations were included in the final analytic cohort. We examined demographic characteristics, clinical factors, resource-use indicators, and DRG classification variables to identify independent predictors of readmission. Model performance was evaluated using multiple complementary metrics, including the area under the receiver operating characteristic curve (AUC), Brier score, Hosmer–Lemeshow goodness-of-fit test, and a decile-based calibration plot.

This study provides comprehensive evidence on short-term readmission risk under a DRG-based payment system and presents an interpretable prediction model that may support risk stratification, guide quality-improvement initiatives, and inform performance monitoring in Chinese tertiary hospitals. By leveraging a large, real-world dataset and rigorous validation measures, our findings aim to contribute to a more nuanced understanding of readmission drivers in the context of China’s ongoing payment reform. Therefore, this study aimed to systematically evaluate 30-day readmission under a DRG-based payment system in a large Chinese tertiary hospital. Specifically, the objectives were to:

1. Estimate the overall 30-day readmission rate;
2. Identify patient- and DRG-level factors associated with readmission;
3. Evaluate the discrimination, calibration, and risk stratification performance of a multivariable logistic regression model based on administrative and DRG data.

## 2 Methods

### 2.1 Study design and data source

This retrospective study used real-world inpatient administrative data from a tertiary hospital in Shanghai, China. The dataset contained 65,215 hospitalization records from January 1, 2023 to December 31, 2024. Each record represented a single inpatient episode and included demographic information, admission and discharge dates, primary diagnosis, principal procedure, DRG code, hospital charges, and length of stay.

### 2.2 Ethics Statement

This study was based on retrospective, fully de-identified administrative inpatient data from a tertiary hospital in Shanghai. No identifiable personal information was accessed. According to institutional policy, analyses of anonymized secondary administrative data are exempt from formal ethics committee review. Therefore, ethical approval and informed consent were not required.

### 2.3 Exclusion criteria and analytic sample

A total of 65,215 inpatient episodes from January 2023 to December 2024 were initially identified.

To ensure complete follow-up for 30-day readmission, all discharges occurring in December 2024 were excluded (*n* = 3,109), yielding 62,106 hospitalizations for descriptive statistics and estimation of the overall readmission rate.

For multivariable modeling, a complete-case analysis was conducted, and 21 records with missing DRG-related variables were removed, resulting in 62,085 admissions included in the final logistic regression model. The proportion of missing data was 0.03% (21/62,106), which is negligible. Given this extremely low level of missingness, the impact of complete-case analysis on model estimates is unlikely to be substantial.

### 2.4 Outcome definition: 30-day readmission

Thirty-day readmission was defined as any subsequent hospitalization recorded in the same hospital within 30 days after discharge from the index admission for the same patient identifier. Because the administrative database did not contain reliable markers distinguishing planned from unplanned admissions, all subsequent admissions within the 30-day window were included in the definition (12).

Hospitalization records were sorted by patient identifier and discharge date. For each admission, the next admission date (if any) was compared with the discharge date to determine whether a readmission occurred within 30 days. A binary variable was created (1 = readmission within 30 days, 0 = no readmission). Readmissions were captured only if they occurred within the same hospital, as cross-institution linkage was not available. Therefore, the estimated readmission rate should be interpreted as an intra-hospital readmission measure rather than a system-wide population rate.

The overall 30-day readmission rate in the complete follow-up cohort (*n* = 62,106) was 13.0% (8,104/62,106).

### 2.5 Variable definitions

#### 2.5.1 Demographic and clinical variables

Age: continuous variable (years).

Length of stay (LOS): number of inpatient days.

Total hospital charges (totcost): continuous variable (Chinese yuan).

Surgery indicator: 1 = surgical, 0 = non-surgical. This variable was used in descriptive comparisons (Table 1) but not included in the multivariable model, as procedural complexity is already reflected in DRG groups, and including both leads to collinearity.

**Table 1:** Baseline characteristics of the study population stratified by 30-day readmission status.

Variable	No readmission (*N* = 54,002)	30-day readmission (*N* = 8,104)	*p* value
Age, years	62.7 (47.3–70.8)	64.1 (52.8–70.8)	<0.001ᵃ
Length of stay, days	4 (2–7)	3 (1–5)	<0.001ᵃ
Total cost, CNY	15,208 (7,373–40,127)	9,634 (5,589–18,203)	<0.001ᵃ
Surgery, *n* (%)	34,915 (64.6%)	2,293 (28.3%)	<0.001ᵇ
Year of discharge, *n* (%)	2023: 26,840 (49.7%) 2024: 27,162 (50.3%)	2023: 4,189 (51.7%) 2024: 3,915 (48.3%)	0.001ᵇ

ᵃ*p* values calculated using Mann–Whitney U test.

ᵇ*p* values calculated using chi-square test.

Values are presented as median (IQR) for continuous variables and *n* (%) for categorical variables.

Total cost is shown in Chinese Yuan (CNY).

Year of discharge: 2023 vs. 2024.

#### 2.5.2 DRG classification

The original DRG code (e.g., FM19) was truncated to the first two characters (e.g., FM) to represent the major DRG category.

A frequency table was generated, and DRG groups with <200 records were merged into a single category labeled “OT,” resulting in 68 categorical levels. Dummy variables were automatically generated during multivariable regression. DRG categories were entered as dummy variables, with the first alphabetical DRG group serving as the reference category in the regression model.

### 2.6 Statistical analysis

#### 2.6.1 Descriptive analysis

Table 1 summarizes patient characteristics in the analytic sample.

Continuous variables were reported using medians and interquartile ranges (IQR) due to right-skewed distributions.

Categorical variables were presented as frequencies and percentages.

#### 2.6.2 Model construction

A single multivariable logistic regression model was fitted using the 62,085 complete cases.

Independent variables included:

Age
Length of stay
Total hospital charges
Year of discharge

DRG major groups (with <200-count groups merged into “OT”). The threshold of 200 was chosen to ensure adequate sample size for stable coefficient estimation while preserving clinical granularity. Sensitivity checks showed that alternative thresholds (e.g., 100 or 300) did not materially change the model’s direction of associations.

Surgery was not included in the multivariable model because procedural complexity is already captured by the DRG classification. Including both variables would introduce collinearity and inflate the instability of coefficients. DRG variables were entered as dummy variables. Multicollinearity was assessed using variance inflation factors (VIFs) derived from an auxiliary linear regression model including all predictors. All VIF values were below 2, indicating no evidence of problematic multicollinearity. To examine the assumption of linearity for length of stay, we additionally conducted a sensitivity analysis by categorizing length of stay into quartiles and re-estimating the model. We selected predictors based on clinical relevance and prior evidence rather than automated selection procedures (e.g., stepwise), in order to avoid overfitting and ensure model interpretability.

Model performance was evaluated through:

Discrimination: AUC
Calibration: Hosmer–Lemeshow (HL) test, Brier score, decile-based calibration curves

#### 2.6.3 Model performance evaluation

Performance was examined using four complementary approaches.

##### 2.6.3.1 Discrimination

Area under the receiver operating characteristic curve (AUC) with 95% CI.

Sensitivity, specificity, and Youden index at the optimal cutoff.

##### 2.6.3.2 Calibration

Hosmer–Lemeshow goodness-of-fit test.

Decile-based calibration plot comparing observed vs. predicted 30-day readmission.

Brier score for overall accuracy.

##### 2.6.3.3 Visualization

See Figures 1–4.

**Figure 1:**
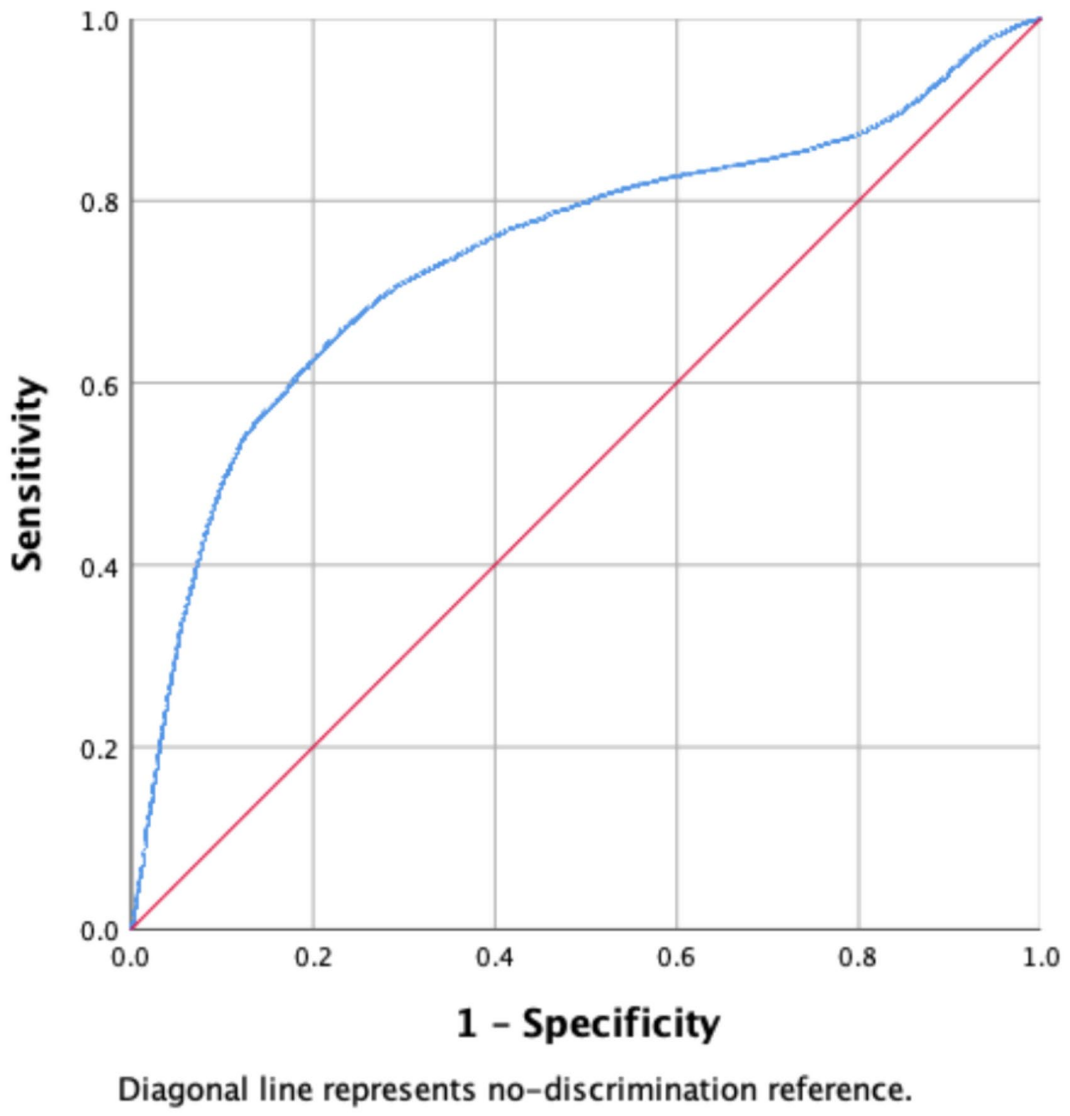
Receiver operating characteristic (ROC) curve of the 30-day readmission prediction model.

**Figure 2:**
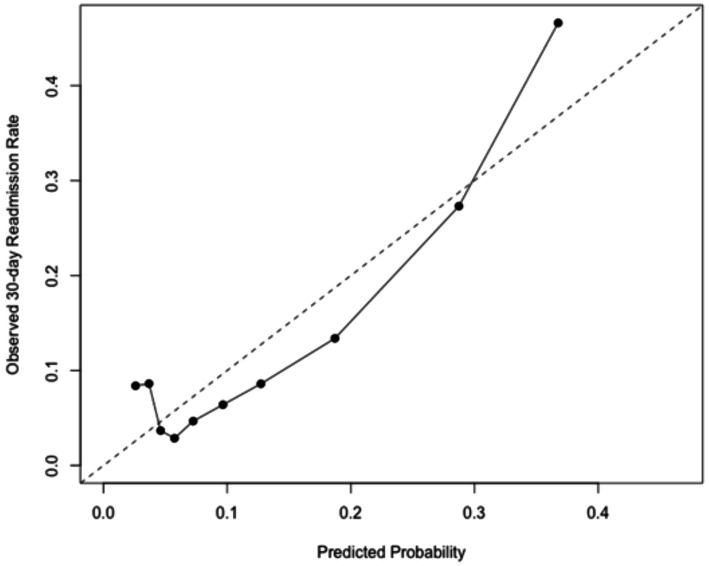
Calibration plot comparing predicted vs. observed 30-day readmission risk.

**Figure 3:**
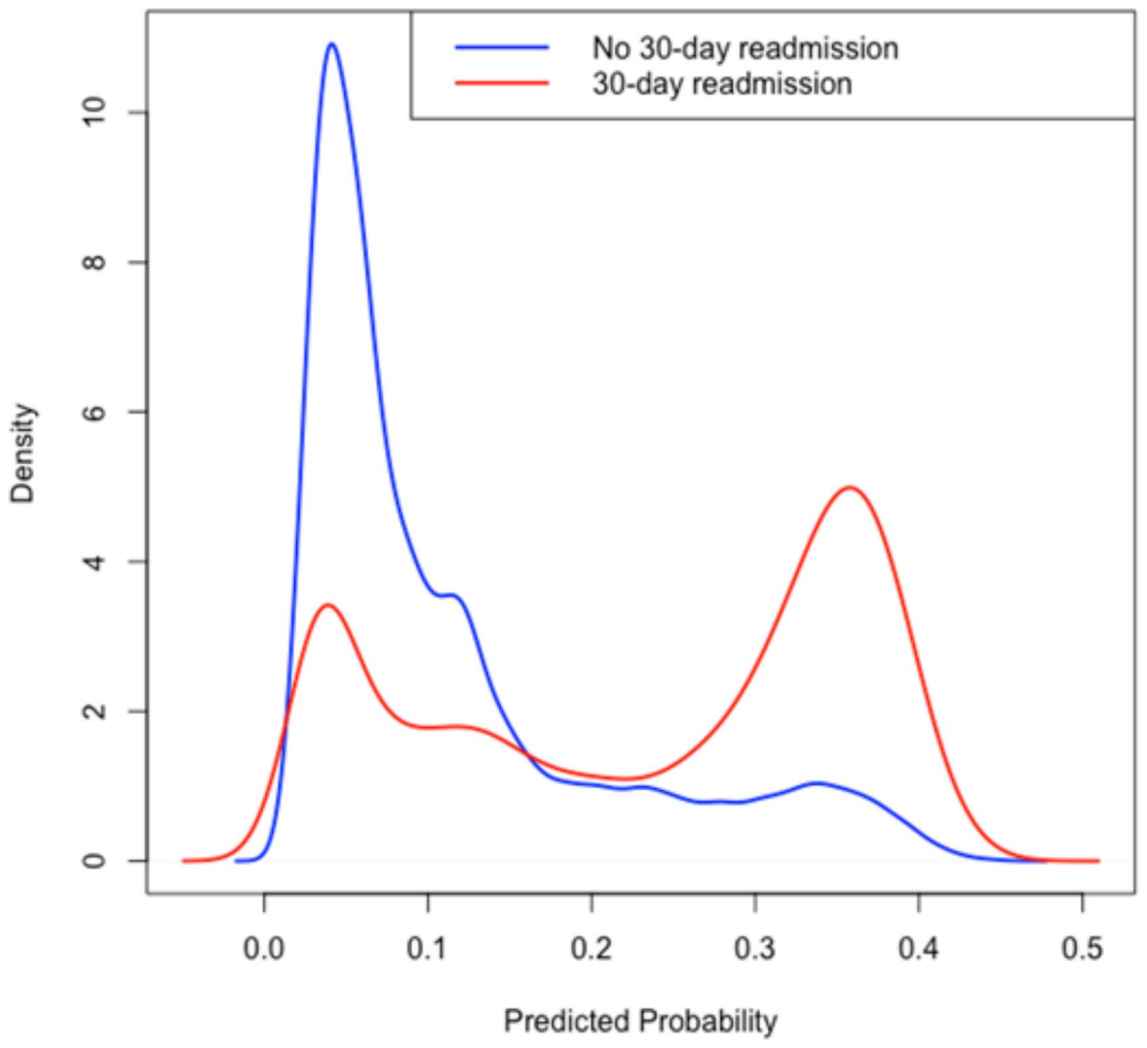
Kernel density distribution of predicted probabilities according to readmission status.

**Figure 4:**
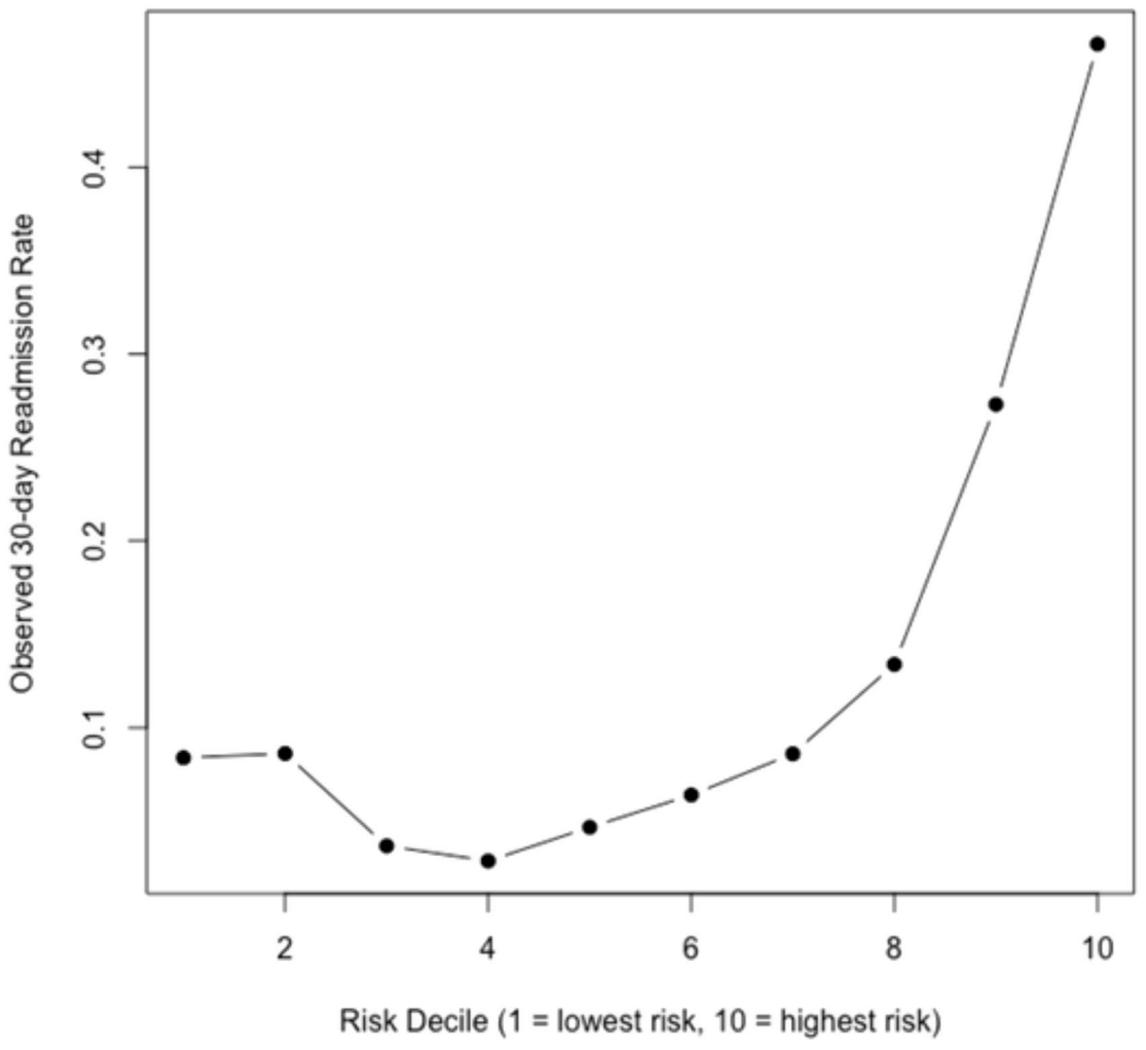
Observed 30-day readmission rate across deciles of predicted risk.

### 2.7 Internal validation

To assess potential overfitting and quantify optimism in model discrimination, we performed internal validation using bootstrap resampling (1,000 iterations). In each bootstrap sample, the model was refitted and evaluated, and optimism was estimated as the mean difference between bootstrap and original AUC values. An optimism-corrected AUC was then calculated. It is reported in the Results section.

All analyses were performed using SPSS 26.0 and R 4.3.3.

A two-sided *p* < 0.05 was considered statistically significant.

## 3 Results

### 3.1 Study population

A total of 62,106 hospitalization records were included in the cohort used to estimate the 30-day readmission rate. Baseline characteristics stratified by readmission status are summarized in Table 1. Among all admissions, 54,002 (87.0%) were not followed by a 30-day readmission, whereas 8,104 (13.0%) resulted in an unplanned readmission within 30 days.

Patients who were readmitted tended to be older, with a median age of 64.1 years (IQR 52.8–70.8) compared with 62.7 years (IQR 47.3–70.8) among those not readmitted. In unadjusted comparisons, the readmission group had shorter lengths of stay (median 3 vs. 4 days) and lower hospitalization costs (median 9,634 vs. 15,208 CNY). They were also less likely to have undergone surgery (28.3% vs. 64.6%). The distribution of discharge year differed significantly between groups (*p* = 0.001).

### 3.2 30-day readmission rate

A total of 8,104 unplanned readmissions occurred within 30 days, corresponding to an overall 30-day readmission rate of 13.0% in the cohort with complete follow-up (8,104/62,106). As shown in Table 1, patients who were readmitted tended to be older and had shorter lengths of stay, lower hospitalization costs, and a lower likelihood of undergoing surgery compared with those who were not readmitted.

### 3.3 Factors associated with 30-day readmission

Multivariable logistic regression results are presented in Table 2. After adjustment for age, length of stay, total cost, year of discharge, and DRG group, longer hospitalization duration was independently associated with a higher likelihood of 30-day readmission (OR 1.016 per day; 95% CI 1.009–1.023; *p* < 0.001). Total hospital cost showed a statistically significant but clinically negligible association with readmission (OR 1.000 per 1 CNY increase, *p* = 0.003). Although statistically detectable in this large sample, the effect size was extremely small, and even substantial increases in cost would correspond to minimal changes in predicted probability. This finding suggests that cost, when modeled at the individual level in its original scale, has limited clinical relevance as an independent predictor. When interpreted over a 1,000 CNY increase, the corresponding odds ratio remained very close to unity, indicating minimal clinical impact despite statistical significance.

**Table 2:** Multivariable logistic regression for 30-day readmission.

Variable	OR	95% CI	*p* value
Age (per 1 year)	1.002	1.000–1.004	0.067
Length of stay (per 1 day)	1.016	1.009–1.023	<0.001
Total cost (per 1 CNY)*	1.000	1.000–1.000	0.003
Year (2024 vs. 2023)	1.004	0.949–1.061	0.9
DRG group*	—	—	<0.001 (overall)

Total cost was modeled in its original unit (1 Chinese Yuan), which produces OR values close to 1 due to the scale.

*DRG category includes 67 groups after merging categories with fewer than 200 observations. DRG variables were entered as dummy variables in the multivariable model.

OR, odds ratio; CI, confidence interval.

Age and discharge year (2024 vs. 2023) were not statistically significant predictors. In contrast, DRG groups showed a strong overall association with readmission risk (global *p* < 0.001), indicating substantial heterogeneity across clinical case-mix categories. To illustrate the magnitude of between-DRG heterogeneity, we present the adjusted odds ratios for the five DRG categories with the highest and lowest readmission risks in Table 3. The highest-risk DRGs demonstrated more than five-fold variation in adjusted odds compared with the reference category, whereas the lowest-risk DRGs showed substantially reduced readmission probabilities. These findings underscore the structural variability in readmission risk across clinical pathways.

**Table 3:** Selected major DRG prefix categories with highest and lowest adjusted odds of 30-day readmission.

DRG prefix	OR	95% CI	*p* value
RE	6.41	4.71–8.73	<0.001
RG	5.19	3.82–7.06	<0.001
RB	5.13	3.45–7.61	<0.001
OZ	2.53	1.74–3.69	<0.001
RU	1.44	1.01–2.04	0.044
KD	0.009	0.002–0.036	<0.001
MJ	0.009	0.001–0.069	<0.001
OC	0.01	0.001–0.071	<0.001
GE	0.02	0.006–0.063	<0.001
ND	0.024	0.006–0.102	<0.001

Although patients who were readmitted had shorter median lengths of stay in unadjusted comparisons (3 vs. 4 days; *p* < 0.001), the direction of association reversed in the fully adjusted model. After accounting for DRG category and other covariates, longer length of stay was associated with higher readmission risk (OR 1.016 per additional day; 95% CI 1.009–1.023; p < 0.001). This pattern suggests that, within the same DRG-defined clinical pathway, prolonged hospitalization likely reflects greater clinical instability, treatment complexity, or unresolved illness, thereby increasing the probability of early readmission.

To assess the linearity assumption for length of stay, a sensitivity analysis was performed by categorizing LOS into quartiles and re-estimating the model. The overall association between LOS categories and 30-day readmission remained statistically significant (global *p* = 0.004). Compared with the reference quartile, the highest LOS quartile demonstrated a statistically significant difference in readmission risk, while intermediate quartiles showed no consistent directional pattern. These findings support the robustness of the primary linear specification and suggest that modeling LOS as a continuous predictor did not materially distort the observed association.

## 4 Model performance

The model’s discrimination, calibration, and risk stratification performance were evaluated using multiple complementary metrics.

### 4.1 Discrimination

The logistic regression model demonstrated acceptable discriminatory ability for predicting 30-day readmission. As shown in Figure 1, the area under the ROC curve (AUC) was 0.743 (95% CI 0.736–0.749), indicating moderate predictive accuracy.

The optimal probability threshold identified using Youden’s index was 0.167, corresponding to a sensitivity of 0.64 and a specificity of 0.79. These results suggest that the model is reasonably effective in distinguishing patients who will be readmitted from those who will not.

Bootstrap internal validation showed minimal optimism. The optimism-corrected AUC (0.743) was nearly identical to the apparent AUC, indicating minimal overfitting and stable internal performance.

### 4.2 Calibration

The Hosmer–Lemeshow (HL) test was statistically significant (*p* < 0.001). However, given the large sample size (*n* = 62,085), even small deviations between observed and predicted probabilities may result in statistical significance. Therefore, HL test results should not be interpreted as evidence of poor calibration in isolation. Visual inspection of the decile-based calibration plot (Figure 2) demonstrated generally good agreement between predicted and observed readmission risks, with only mild deviation in the highest-risk groups. The Brier score was 0.098, indicating good overall accuracy of predicted probabilities.

### 4.3 Distribution of predicted risk

The distribution of predicted probabilities stratified by readmission status is shown in Figure 3. Patients who were readmitted exhibited a visibly higher density peak at greater predicted probabilities, indicating that the model effectively assigns higher risk scores to individuals who ultimately experienced a 30-day readmission.

### 4.4 Risk stratification

To assess clinical risk stratification capability, predicted probabilities were grouped into deciles. Observed 30-day readmission rates rose progressively across deciles (Figure 4), increasing from approximately 8% in the lowest decile to over 45% in the highest decile. This monotonic pattern demonstrates strong stratification performance and supports the potential utility of the model for identifying high-risk patients.

## 5 Discussion

This study provides one of the most comprehensive examinations of 30-day readmission patterns in a Chinese tertiary hospital operating under a maturing DRG-based payment system. It is important to emphasize that the present findings represent statistical associations observed within a single institutional context and should not be interpreted as evidence of direct causal effects of DRG-based payment on readmission risk. Drawing on more than 62,000 inpatient encounters and applying a rigorous analytic framework that integrates multivariable regression, discrimination and calibration assessment, and risk stratification analyses, we identified patient-level predictors and system-level implications of early readmission. Our findings add to a small but expanding body of evidence evaluating the performance and unintended consequences of DRG-based reforms in China. They also situate the Chinese experience within broader global debates surrounding readmission penalties, hospital efficiency, care coordination, and the shifting role of acute-care institutions in value-based health systems. These findings should therefore be interpreted as observational associations within a single institutional context rather than causal evidence of DRG-induced behavioral change.

### 5.1 Principal findings

The overall 30-day readmission rate of 13.0% in this study is broadly consistent with international benchmarks for mixed-condition adult populations, although it remains lower than the condition-specific rates commonly reported in Medicare data for high-risk diagnoses such as heart failure or pneumonia. Age and length of stay emerged as the primary predictors of early readmission, aligning with patterns documented in multiple OECD health systems (13), where older patients—often with multimorbidity, polypharmacy, frailty, or functional decline—face a heightened likelihood of clinical deterioration shortly after discharge (14).

A key finding concerns the complex role of length of stay. In crude comparisons, patients who were readmitted had shorter hospitalizations, echoing concerns raised in the literature that prospective payment systems may be associated with organizational pressures toward earlier discharge timing, although our findings cannot establish causal relationships (15). However, after adjusting for DRG category and other covariates, the association reversed: within any given DRG-defined clinical pathway, longer stays were associated with a higher readmission risk. This apparent contradiction underscores that length of stay captures different dimensions of severity at different analytic levels. Across DRGs (“between-group”), shorter stays may reflect premature discharge. Within DRGs (“within-group”), longer stays likely signal unresolved clinical instability, treatment delays, postoperative complications, or complex therapeutic needs—factors that naturally increase the likelihood of returning within 30 days. Taken together, these findings should be interpreted as associations observed within a specific institutional context rather than as direct causal effects of DRG payment.

Hospitalization cost showed a statistically significant but clinically negligible association with readmission. Given the very small marginal effect observed, cost should not be interpreted as a meaningful independent driver of readmission risk, but rather as a potential proxy for unmeasured clinical complexity. Although the odds ratio per 1 CNY increase was statistically significant, the magnitude of effect was extremely small. Even when scaled to a 1,000 CNY increase, the change in predicted probability remained minimal, underscoring that statistical significance does not necessarily imply meaningful clinical or policy relevance. This is expected in a DRG-based system, where payments—not actual expenditures—drive hospital financial behavior. Cost therefore appears to offer limited clinical interpretability within this administrative framework, and our findings reinforce arguments that quality monitoring under DRG reform should shift away from cost-centered indicators toward patient-centered outcomes such as avoidable readmission, discharge safety, and care-transition quality.

The inclusion of DRG groupings was associated with improved model fit and discrimination. At the meantime, it revealed marked heterogeneity in readmission risk across clinical categories. These adjusted estimates reflect comparisons relative to the reference DRG group rather than absolute measures of intrinsic clinical severity. High-risk DRGs—including those involving acute medical instability or surgical episodes with intrinsic susceptibility to complications—displayed notably higher adjusted odds of readmission, whereas stable chronic or elective-procedural DRGs showed substantially lower risk. This pattern closely mirrors findings reported in the United States, England, Germany, and South Korea, indicating that variability in readmission risk is a structural feature of modern hospital systems rather than an artifact of local practice. The modest calibration challenges observed in our model (as reflected in the Hosmer–Lemeshow test) further emphasize the inherent difficulty of fitting a single universal prediction model across highly heterogeneous DRG populations.

### 5.2 Comparison with existing international evidence

Extensive international evidence has documented that DRG-based prospective payment systems have been associated with improvements in hospital efficiency and cost containment, may also generate unintended consequences for care continuity and post-discharge outcomes (15, 16). Experience from the United States, where the Hospital Readmission Reduction Program (HRRP) has linked financial penalties to early readmissions since 2012, illustrates both the potential and the limitations of readmission-based quality incentives (17, 18). HRRP has been associated with measurable reductions in readmission rates for targeted conditions (19), but subsequent evaluations raised concerns regarding risk-shifting behaviors, including increased use of emergency observation units, delayed readmissions beyond the 30-day window, and challenges in adequately adjusting for socioeconomic and clinical complexity (20). In England, the National Health Service initially piloted readmission penalties within the Payment by Results (PbR) framework but gradually moved toward blended payment models and integrated care pathways after evidence accumulated that punitive financial mechanisms alone were insufficient to address fragmentation between inpatient and community services (21). Similarly, health systems in Germany and Australia monitor readmissions closely but rely less on direct penalties, emphasizing instead the importance of standardized care pathways, strengthened transitional care, and collaboration between hospitals and primary care providers (22, 23). Across OECD countries, a consistent narrative has emerged: while DRG payment can improve efficiency, its influence on post-acute outcomes depends on the degree to which health systems support coordinated care and mitigate incentives for premature discharge.

China’s implementation of DRG-based payment differs from these international systems in both timing and policy orientation. Rather than introducing explicit readmission penalties, China’s reform has focused on standardizing clinical pathways, controlling inpatient expenditure growth, and establishing budget accountability across hospitals (24). In this context, our findings highlight how early readmission can serve as a sensitive indicator of the tensions inherent in a maturing DRG environment. The association between shorter length of stay and elevated unadjusted readmission risk suggests that hospitals may be operating under meaningful time and efficiency pressures. At the same time, the substantial variation in readmission across DRG categories underscores the need for condition-sensitive policy design, aligning closely with international experiences that emphasize differential management of high-risk diagnostic groups.

Overall, the patterns observed in this study reinforce global lessons: DRG-based payment reforms must be paired with robust transitional care, adequate risk adjustment, and system-level coordination to avoid unintended quality trade-offs. China’s ongoing reform presents a critical opportunity to incorporate these lessons early in the development of its national DRG framework.

### 5.3 Implications for DRG-based payment reform in China

The implications of our findings extend beyond simple risk prediction and touch on the broader architecture of China’s ongoing DRG-based payment reforms. First, our results highlight the value of incorporating readmission indicators into routine quality monitoring within DRG systems. Because DRGs primarily incentivize cost containment and efficiency, hospitals may unintentionally shorten inpatient stays in ways that are associated with higher observed readmission rates. A risk-adjusted readmission measure—calculated either across all DRGs or within specific high-risk groups—could serve as a counterbalancing metric. Integrating such indicators into performance dashboards or payment adjustments would help ensure that efficiency gains do not come at the expense of clinical safety. However, these policy considerations should be interpreted in light of the study’s single-center design, and broader national implementation would require validation across diverse hospital settings and regional DRG contexts.

Second, the pronounced variation in readmission risk across major DRG categories suggests that China would benefit from differentiated DRG-level policy levers, rather than uniform rules. In mature DRG systems such as Germany and Australia, high-severity or clinically volatile DRGs are often granted extended length-of-stay thresholds, enhanced payment flexibility, or supplemental quality monitoring (25, 26). Adopting similar nuance in Chinese DRGs—such as tailoring outlier policies or providing higher case weights for clinically unstable DRGs—may help reduce pressure toward premature discharge and better align reimbursement with patient needs.

Third, our findings underscore the need to strengthen post-acute continuity of care, given that a considerable proportion of readmissions stem from failures in transitional care, medication management, and outpatient follow-up. International evidence has consistently shown that structured transition programs, telehealth-assisted monitoring, primary care linkage, and community-based rehabilitation can effectively lower readmission rates (27, 28). In China, where digital infrastructure is rapidly advancing and community health centers are expanding under hierarchical medical reform, integrating such transitional care models into DRG payment rules may yield substantial quality and efficiency benefits.

Fourth, the calibration limitations of our prediction model reveal the constraints of relying solely on administrative data for risk adjustment. Important predictors—frailty, functional status, socioeconomic vulnerability, caregiver availability, and health literacy—remain unmeasured in most Chinese hospital information systems. As China develops a unified national health information platform, incorporating structured clinical data, medication histories, and patient-reported outcomes will be crucial for improving risk adjustment and building fairer benchmarking frameworks under DRG payment.

Finally, our findings reinforce that readmission risk is not merely a reflection of inpatient decision-making but rather a system-level indicator shaped by broader care fragmentation, uneven access to primary care, and limited long-term care resources. Policymakers should therefore interpret readmission metrics as part of an integrated ecosystem of incentives and constraints, recognizing that hospitals alone cannot rectify upstream social and outpatient challenges.

Operationally, the proposed model could be integrated into hospital information systems as part of routine quality monitoring dashboards. For example, predicted readmission risk scores could be generated at discharge to flag high-risk patients for enhanced transitional care planning, medication reconciliation, or early follow-up scheduling. At the system level, payers could use risk-adjusted readmission benchmarks within specific high-risk DRG categories to identify outlier institutions and guide targeted quality-improvement interventions rather than applying uniform penalties. Such applications should prioritize risk adjustment, transparency, and supportive feedback mechanisms, ensuring that predictive analytics are used to improve care coordination rather than to impose simplistic performance sanctions.

### 5.4 Strengths and limitations

This study has several notable strengths. It draws on a large, real-world administrative dataset comprising more than 62,000 inpatient encounters from a high-volume tertiary hospital, enabling a granular assessment of readmission patterns across diverse DRG categories. The analytic strategy incorporated multiple complementary performance metrics—including discrimination, calibration, and risk stratification—providing a comprehensive evaluation rarely seen in existing Chinese DRG literature. Furthermore, by explicitly comparing crude and adjusted findings for length of stay, the study offers nuanced insights into how DRG payment structures shape clinical decision-making at both the between-DRG and within-DRG levels.

Nonetheless, several limitations must be acknowledged. First, the analysis relied exclusively on hospital administrative data, which lack important clinical detail such as comorbidity scores, physiological indicators, disease severity, frailty status, and functional capacity. These omissions may have led to residual confounding and restricted the ability of the model to fully capture patient complexity. Second, because readmissions were identified only within the same hospital, cross-institution readmissions were missed. International evidence suggests that 10–30% of all unplanned readmissions may occur at different facilities (29), which implies that our readmission rate is likely an underestimate. Third, the absence of outpatient, pharmacy, and primary-care data limits our ability to account for transitional care processes and post-discharge adherence, both of which are major contributors to early readmissions. Fourth, as a single-center study conducted within a specific institutional and regional context, generalizability is limited; patterns observed in Shanghai may differ substantially from those in less-resourced provinces or hospitals with different DRG maturity levels. Moreover, because planned and unplanned readmissions could not be distinguished in the data, the reported readmission rate may be upwardly biased, and some degree of nondifferential misclassification is possible. Finally, although complete-case analysis was applied, the proportion of missing data was extremely small (0.03%), reducing the likelihood of meaningful bias. Nevertheless, if missingness was related to documentation practices or unobserved severity, minor selection effects cannot be entirely excluded.

### 5.5 Future directions

Future research should build upon these findings by integrating more granular clinical and patient-reported data to improve risk adjustment and enhance the interpretability of readmission models in DRG environments. Linking inpatient data with primary care, pharmacy claims, and community health records would allow for a more complete characterization of transitional care gaps and post-discharge trajectories. Prospective cohort studies, particularly those incorporating frailty measures, functional assessments, biomarkers, and socioeconomic indicators, could provide deeper insight into mechanisms underlying early readmission. Additionally, multi-center investigations across provinces with different DRG implementation stages would help assess external generalizability and identify region-specific policy levers. As China advances toward a national health information infrastructure, future studies should explore machine-learning approaches and dynamic risk monitoring that support real-time, individualized prediction. Evaluating the impact of targeted interventions—such as structured discharge planning, telehealth follow-up, and community-based care coordination—will also be critical for translating predictive insights into measurable improvements in patient outcomes.

## 6 Conclusion

In conclusion, this single-center real-world study provides robust evidence on 30-day hospital readmissions within a DRG-based payment environment in China. We identified substantial heterogeneity in readmission risk across DRG categories and demonstrated that length of stay and patient complexity remain important correlates of early readmission even under prospective payment.

Although the model achieved moderate discrimination and reasonable calibration, its performance also reflects the inherent constraints of administrative data–based risk adjustment. These findings support the use of risk-adjusted readmission indicators as complementary quality-monitoring tools within DRG systems, while emphasizing the importance of transitional care and system-level coordination to ensure that efficiency gains do not compromise patient safety.

## Data Availability

The data analyzed in this study is subject to the following licenses/restrictions: the dataset contains individual-level inpatient administrative records obtained from a tertiary hospital in China. Due to patient privacy protections, institutional data governance policies, and ethical approval restrictions, the data are not publicly available. Requests to access these datasets should be directed to shaowei@lnvcm.edu.cn.

## References

[1] Glans M Kragh Ekstam A Jakobsson U Bondesson Å Midlöv P. Risk factors for hospital readmission in older adults within 30 days of discharge – a comparative retrospective study. BMC Geriatr. (2020) 20:467. doi: 10.1186/s12877-020-01867-3, 33176721 PMC7659222

[2] Ebhohon E Khoshbin K Shaka H. Rates and predictors of 30-day hospital readmissions in adults for drug-induced acute pancreatitis: A retrospective study from the United States National Readmission Database. J Gastroenterol Hepatol. (2023) 38:1277–82. doi: 10.1111/jgh.16177, 36914611

[3] Fu BQ Zhong CC Wong CH Ho FF Nilsen P Hung CT . Barriers and facilitators to implementing interventions for reducing avoidable hospital readmission: systematic review of qualitative studies. Int J Health Policy Manag. (2023) 12:7089. doi: 10.34172/ijhpm.2023.7089, 37579466 PMC10125127

[4] King L Harrington A Nicholls S Thornton K Tanner E. Towards reduction of preventable hospital readmission: Older people and family members' views on planned self-management of care at home. J Clin Nurs. (2023) 32:4599–613. doi: 10.1111/jocn.16492, 35974684

[5] Cram P Wachter RM Landon BE. Readmission Reduction as a Hospital Quality Measure: Time to Move on to More Pressing Concerns? JAMA. (2022) 328:1589–90. doi: 10.1001/jama.2022.18305, 36201190

[6] Xiang X Dong L Qi M Wang H. How does diagnosis-related group payment impact the health care received by rural residents? Lessons learned from China. Public Health. (2024) 232:68–73. doi: 10.1016/j.puhe.2024.04.021, 38749150

[7] Luo M Li H Li R Wu Y Lan Y Xie S. The impact of DRG payment reform on inpatient costs for different surgery types: an empirical analysis based on Chinese tertiary hospitals. Front Public Health, (2025), 13:1563204. doi: 10.3389/fpubh.2025.1563204 40529694 PMC12170532

[8] Ren S Yang L Du J He M Shen B. DRGKB: a knowledgebase of worldwide diagnosis-related groups’ practices for comparison, evaluation and knowledge-guided application. Database. (2024) 2024:baae046. doi: 10.1093/database/baae046, 38843311 PMC11155695

[9] Huang Y Li W Macheret F Gabriel RA Ohno-Machado L. A tutorial on calibration measurements and calibration models for clinical prediction models. J Am Med Inform Assoc. (2020) 27:621–33. doi: 10.1093/jamia/ocz228, 32106284 PMC7075534

[10] Li Q Fan X Jian W. Impact of diagnosis-related-group (DRG) payment on variation in hospitalization expenditure: evidence from China. BMC Health Serv Res. (2023) 23:688. doi: 10.1186/s12913-023-09686-z, 37355657 PMC10290781

[11] Li L Yang W Tang X Zeng S Liu X Dong S. The impacts of the diagnosis-related group payment reform on hospitalization-related medical expenses: evidence from China. Health Econ Rev. (2025) 15:1–10. doi: 10.1186/s13561-025-00687-8, 41186830 PMC12584354

[12] Xu X Gong K Hong L Yu X Tu H Lan Y . The burden and predictors of 30-day unplanned readmission in patients with acute liver failure: a national representative database study. BMC Gastroenterol. (2024) 24:153. doi: 10.1186/s12876-024-03249-0, 38702642 PMC11067096

[13] Bopche R Gustad LT Afset JE Ehrnström B Damås JK Nytrø Ø. In-hospital mortality, readmission, and prolonged length of stay risk prediction leveraging historical electronic patient records. JAMIA Open. (2024) 7:ooae074. doi: 10.1093/jamiaopen/ooae074 39282081 PMC11401612

[14] Hoel RW Giddings Connolly RM Takahashi PY. Polypharmacy Management in Older Patients. Mayo Clin Proc. (2021) 96:242–56. doi: 10.1016/j.mayocp.2020.06.012, 33413822

[15] Meng Z Hui W Cai Y Liu J Wu H. The effects of DRGs-based payment compared with cost-based payment on inpatient healthcare utilization: A systematic review and meta-analysis. Health Policy. (2020) 124:359–67. doi: 10.1016/j.healthpol.2020.01.007, 32001043

[16] Dong X Wu J. Does DRG-based payment lead to unintended effects on care quality? A case under global budget with price adjustment in China. BMC Health Serv Res. (2025) 25:1448. doi: 10.1186/s12913-025-13625-5, 41194069 PMC12590802

[17] Lahijanian B Alvarado M. Care strategies for reducing hospital readmissions using stochastic programming. Healthcare. (2021) 9:940. doi: 10.3390/healthcare9080940, 34442079 PMC8393874

[18] Ody C Msall L Dafny LS Grabowski DC Cutler DM. Decreases in readmissions credited to Medicare’s program to reduce hospital readmissions have been overstated. Health Aff. (2019) 38:36–43. doi: 10.1377/hlthaff.2018.05178, 30615522

[19] Gupta A Fonarow GC. The hospital readmissions reduction program. JACC. (2018) 6:607–9. doi: 10.1016/j.jchf.2018.02.012, 29957194

[20] Wright B Parrish C Basu A Joynt Maddox KE Liao JM Sabbatini AK. Medicare's hospital readmissions reduction program and the rise in observation stays. Health Serv Res. (2023) 58:554–9. doi: 10.1111/1475-6773.14142, 36755372 PMC10154161

[21] Stokes J Struckmann V Kristensen SR Fuchs S van Ginneken E Tsiachristas A . Towards incentivising integration: A typology of payments for integrated care. Health Policy. (2018) 122:963–9. doi: 10.1016/j.healthpol.2018.07.003, 30033204

[22] Busse R Stahl J. Integrated care experiences and outcomes in Germany, the Netherlands, and England. Health Aff (Millwood). (2014) 33:1549–58. doi: 10.1377/hlthaff.2014.0419, 25201659

[23] Aaron MB Kerrissey M Novikov Z Tietschert MV Scherling A Bahadurzada H . The association between care integration and care quality. Health Serv Res. (2024) 59:e14214. doi: 10.1111/1475-6773.14214, 37605429 PMC11622280

[24] Liu R Shi J Yang B Jin C Sun P Wu L . Charting a path forward: policy analysis of China's evolved DRG-based hospital payment system. Int Health. (2017) 9:317–24. doi: 10.1093/inthealth/ihx030, 28911128 PMC5881274

[25] Jackson T Dimitropoulos V Madden R Gillett S. Australian diagnosis related groups: Drivers of complexity adjustment. Health Policy. (2015) 119:1433–41. doi: 10.1016/j.healthpol.2015.09.011, 26521013

[26] Quentin W Stephani V Berenson RA Bilde L Grasic K Sikkut R . How Denmark, England, Estonia, France, Germany, and the USA Pay for Variable, Specialized and Low Volume Care: A Cross-country Comparison of In-patient Payment Systems. Int J Health Policy Manag. (2022) 11:2940–50. doi: 10.34172/ijhpm.2022.6536, 35569000 PMC10105175

[27] Albritton JA Booth G Kugley S Reddy S Coker-Schwimmer M Fujita M . Audio-Based Care for Managing Chronic Conditions in Adults: A Systematic Review. Med Care. (2025) 63:164–82. doi: 10.1097/MLR.0000000000002097, 39791849 PMC11708983

[28] Savira F Gupta A Gilbert C Huggins CE Browning C Chapman W . Virtual care initiatives for older adults in Australia: scoping review. J Med Internet Res. (2023) 25:e38081. doi: 10.2196/38081, 36652291 PMC9892987

[29] Amritphale A Fonarow GC Amritphale N Omar B Crook ED. All-cause unplanned readmissions in the United States: insights from the Nationwide Readmission Database. Intern Med J. (2023) 53:262–70. doi: 10.1111/imj.15581, 34633136

